# Challenging cases of adherent periarterial vein during subinguinal Fisch technique and subinguinal micro-varicocelecotmy and sclerotherapy: a prospective comparative study

**DOI:** 10.1186/s12610-025-00250-9

**Published:** 2025-02-04

**Authors:** Amr Elahwany, Nashaat Nabil, Sameh Fayek GamalEl Din, Ahmed Raef Sadek, Ahmed Ewais Sayed, Ahmed Ragab

**Affiliations:** 1 Department of Andrology, Sexology and STDs, Kasr Alainy Faculty of Medicine, Cairo University, Cairo, Egypt; 2 Nile Center for IVF, Cairo, Egypt; 3 Department of Andrology, Sexology and STDs, Faculty of Medicine, Beni-Suef University, Beni Suef, Egypt

**Keywords:** Adherent periarterial vein, Sperm progressive motility, Colpi sclerotherapy, Fisch technique, Microsurgical varicocelectomy, Veine périartérielle adhérente, Motilité progressive des Spermatozoïdes, Sclérothérapie de Colpi, Technique de Fisch, Varicocélectomie microchirurgicale

## Abstract

**Background:**

To the best of our knowledge, there is a gap in the review of literature about the most suitable varicocelectomy technique in isolating and ligating adherent periarterial vein(s). Consequently, leaving the artery intact or ligating it together with the adherent vein may pose a challenge. We conducted a comparative prospective study to assess the outcomes of the three techniques namely Fisch, sclerotherapy and microvaricoclectomy (MSV).

**Results:**

The patients who underwent MSV showed the longest operative time (66.29 ± 2.78 min), followed by Fisch technique (56.94 ± 3.07 min) then sclerotherapy (55.45 ± 1.99). Thus, the difference in the operative time between the three techniques was statistically significant (P < 0.001). Regarding the postoperative right vein diameter, MSV group showed the largest diameter (2.14 ± 0.15 mm), followed by Fisch technique (2.13 ± 0.15 mm) then sclerotherapy (1.75 ± 0.42 mm). Regarding the postoperative left vein diameter, MSV group showed again the largest diameter (2.17 ± 0.21 mm), followed by Fisch technique (2.14 ± 0.15 mm) then sclerotherapy (1.75 ± 0.42 mm). Moreover, the patients who underwent sclerotherapy showed the highest postoperative progressive sperm motility percent (25.27 ± 4.00%), followed by Fisch technique (21.56 ± 7.30%) then MSV group (19.85 ± 6.33%). Post hoc pair wise comparisons revealed that sclerotherapy and Fisch technique had a significantly higher effectiveness in reducing operative time than MSV. Additionally, it revealed that sclerotherapy technique had a significantly higher effectiveness in reducing postoperative vein diameters measurement than MSV and Fisch technique. Post hoc pair wise comparisons revealed that sclerotherapy technique had a significantly higher effectiveness in improving the postoperative progressive sperm motility percent than MSV.

Patients who underwent the 3 techniques demonstrated statistically significant differences between baseline and post-operative vein diameter, reflux duration, sperm concentration, progressive sperm motility, progressive motile count/ejaculate and sperm abnormal forms.

**Conclusion:**

The 3 techniques showed significant improvement in the semen parameters after 3 months in the studied patients. However, sclerotherapy technique showed a significantly higher effectiveness in improving the postoperative progressive sperm motility percent compared to MSV.

## Résumé

**Contexte:**

À notre connaissance, il existe une lacune dans la littérature sur la technique de varicocélectomie la plus appropriée pour isoler et ligaturer la ou les veines adhérentes péri artérielles. Par conséquent, laisser l’artère intacte ou la ligaturer avec la veine adhérente peut constituer un défi. Nous avons mené une étude prospective comparative pour évaluer les résultats des trois techniques, à savoir la méthode de Fisch, la sclérothérapie et la microchirurgie de la varicocèle (MCV).

**Résultats:**

Les patients qui ont subi une MCV ont montré le plus long temps opératoire (66,3 ± 2,8 min), suivi de la technique de Fisch (56,9 ± 3,1 min) puis de la sclérothérapie (55,5 ± 1,9). La différence de temps opératoire entre les trois techniques était, ainsi, statistiquement significative (p < 0,001). En ce qui concerne le diamètre postopératoire de la veine droite, le groupe MCV présentait le plus grand diamètre (2,14 ± 0,15 mm), suivi de la technique de Fisch (2,13 ± 0,15 mm) puis de la sclérothérapie (1,75 ± 0,42 mm). En ce qui concerne le diamètre postopératoire de la veine gauche, le groupe MCV a de nouveau montré le plus grand diamètre (2,17 ± 0,21 mm), suivi de la technique de Fisch (2,14 ± 0,15 mm) puis de la sclérothérapie (1,75 ± 0,42 mm). De plus, les patients qui ont subi une sclérothérapie ont montré le pourcentage le plus élevé de motilité progressive des spermatozoïdes en postopératoire (25,3 ± 4,0%), suivi de la technique Fisch (21,6 ± 7,3%) puis de la MCV (19,8 ± 6,3%). Les comparaisons post-hoc par paires ont révélé que la sclérothérapie et la technique de Fisch avaient une efficacité significativement plus élevée pour réduire le temps opératoire que le MCV. De plus, la technique de sclérothérapie avait une efficacité significativement plus élevée pour réduire la mesure des diamètres veineux postopératoires que la MCV et la technique de Fisch. Les comparaisons post-hoc par paires ont montré que la technique de sclérothérapie avait une efficacité significativement plus élevée pour améliorer le pourcentage de motilité progressive des spermatozoïdes en postopératoire que la MCV.

**Conclusion:**

Les 3 techniques ont montré une amélioration significative des paramètres du sperme après 3 mois. Cependant, la technique de sclérothérapie a montré une efficacité significativement plus élevée dans l’amélioration du pourcentage de motilité progressive des spermatozoïdes postopératoire par rapport à la MCV.

## Introduction

Varicocele (Vx) is defined as an abnormal dilatation and tortuosity of the internal spermatic veins found within the pampiniform plexus [1]. Clinically; Vx can be accompanied by scrotal pain, discomfort and progressive testicular hypofunction [2]. Although the specific pathophysiology leading to impaired spermatogenesis is not fully understood, numerous studies had shown varicocelectomy to be effective in improving pregnancy rate through improvements in semen quality, especially with regards to sperm motility and concentration [3]. The impact of Vx on semen accounts for 21%–41% of men with primary infertility and 75%–81% of men with secondary infertility [4], being among the correctable causes of male infertility [5]. The ultimate goals of varicocelectomy include occlusion of the refluxing variceal veins draining the testis, while hardly aiming at selective sparing of arterial inflow and lymphatic drainage to improve testicular function [6]. Surgical treatment of varicocele improves sperm concentration, motility, and morphology [7]. Also, sclerotherapy significantly improves sperm count, motility, and morphology [2]. However; the advantages of each surgical method are still controversial [8]. Marmar et al. (1985) presented the first microsurgical varicocelectomy (MSV) [9]. In 1994, Marmar & Kim demonstrated that the recurrence of a palpable Vx was 0.82% based on the total number of the procedures that were performed on these men [10]. Previous studies showed that MSV had a better outcome, a higher pregnancy rate and a lower incidence of complications [11]. Sclerotherapy had been introduced as a less invasive option for occlusion of the venous vessels [12]. Colpi et al. (2006) utilized a subinguinal approach combined with sclerotherapy alone for occlusion of the venous vessels by a modification of Marmar and Kim (1994) technique [10, 13]. This variant is simpler and has lower management costs, as neither an optical microscope nor fluoroscopic control is needed [13].

At the subinguinal level, testicular arteries are more frequently surrounded by a network of adherent veins than at the inguinal level, making dissection and separation of the attached vessels more difficult with the subinguinal approach than at a high level [14]. Fisch et al. (2004) introduced a short timed artery-sparing and lymphatic-sparing varicocelectomy technique for difficult to isolate periarterial veins as a modification of the classic MSV [15]. To the best of our knowledge, there is a gap in the review of literature about the most suitable varicocelectomy technique in isolating and ligating adherent periarterial vein(s). Consequently, leaving the artery intact or ligating it together with the adherent vein may pose a challenge. Thus, we aimed in the current study to assess and compare the outcomes of the three techniques namely Fisch, sclerotherapy and MSV employed in the subinguinal repair of Vx, particularly in challenging cases with adherent periarterial vein(s).

## Material and methods

**Fig. 1:**
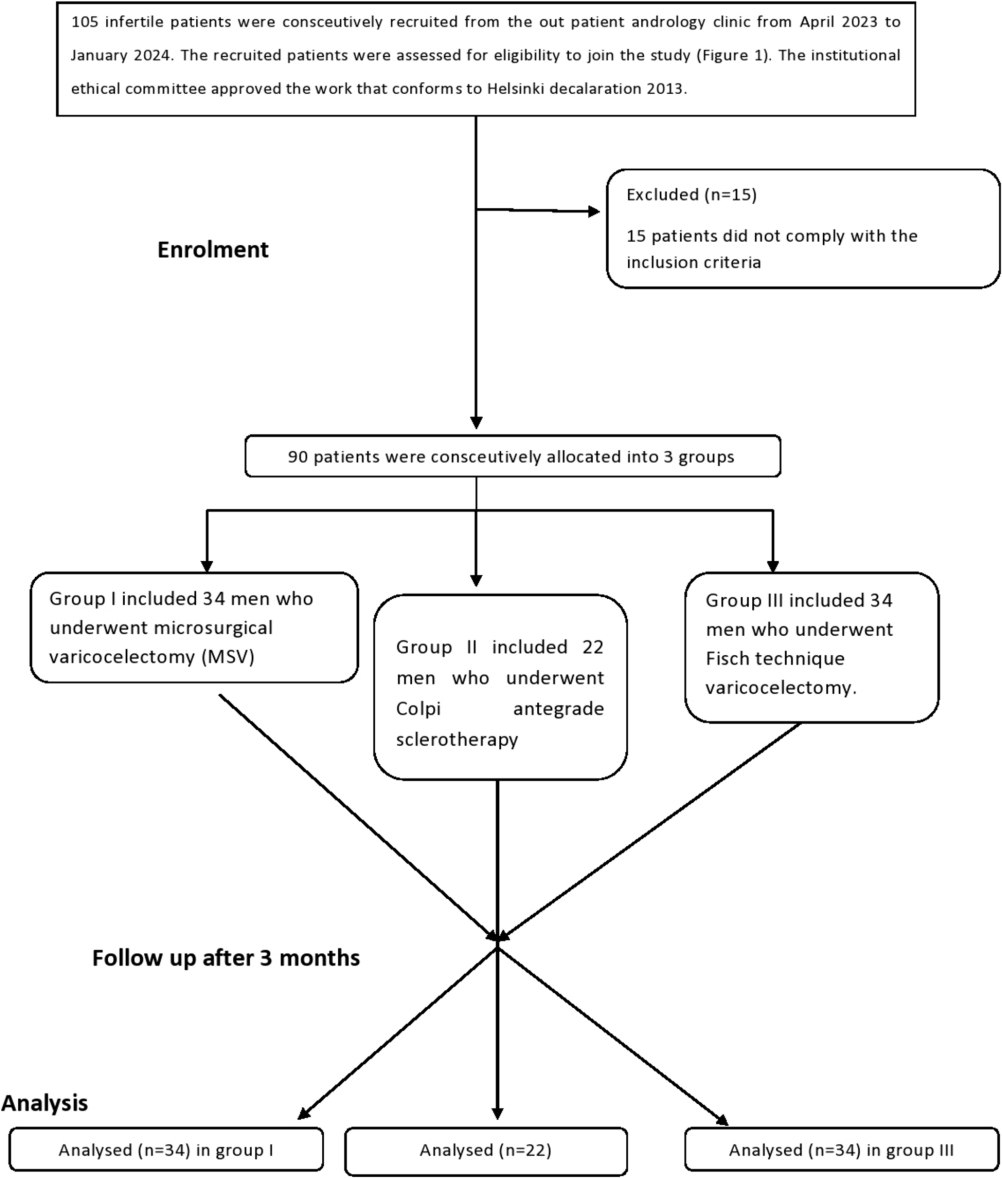
Flowchart of the study

This prospective comparative study was held at the andrology department, Beni-suef university hospital from April 2023 to January 2024 (Fig. 1). Eligible participants were counselled pre-operatively, and were given written informed consent to perform the surgery, according to the regulations mandated by the Research Ethical Committee of Beni-suef Faculty of Medicine which conform to Helsinki declaration 2013 (IORG0006240) [16]. All participants signed an informed consent prior to enrolment about the nature of the study with the necessity to undergo varicocelectomy to evaluate different techniques on their semen parameters and the potential postoperative complications as well.

### Inclusion criteria

Intra-operative identification of adherent or difficult-to-isolate periarterial vein within the pampiniform plexus at the sub-inguinal level was the main prerequisite to join the current study. Additionally, the included patients were infertile men with palpable Vx and abnormal sperm parameters according to WHO (2010) guidelines [17] whose female partners being 35 years or younger and healthy.

### Exclusion criteria

Cases of secondary Vx, men with semen volume < 1.5 ml, azoospermia or cryptozoospermia, untreated urogenital tract infection, cases of immnulogical infertility, cases of testicular tumors, cases of uncontrolled or severe systemic illnesses or cases unfit for surgery were excluded. Also, cases who suffered from hormonal imbalance were excluded.

All patients were subjected to the following:

The patients were consecutively allocated into the following groups:

Group I included 34 men who underwent MSV. Group II included 22 men who underwent Colpi antegrade sclerotherapy. Group III included 34 men who underwent Fisch technique varicocelectomy. Medical and surgical histories were obtained. General and genital examinations were conducted. Semen analyses were pre-operatively and 3 months post-operatively obtained according to the WHO, 2010 guidelines [17]. Furthermore, Vx was diagnosed according to the guidelines set by the European Society of Urogenital Radiology Scrotal and Penile Imaging Working Group [18]. Pre-operative grey scale, color Doppler US and spectral Doppler analysis [Mindray DP-30 device] were performed bilaterally with and without Valsalva, while standing and supine.

**Fig. 2:**
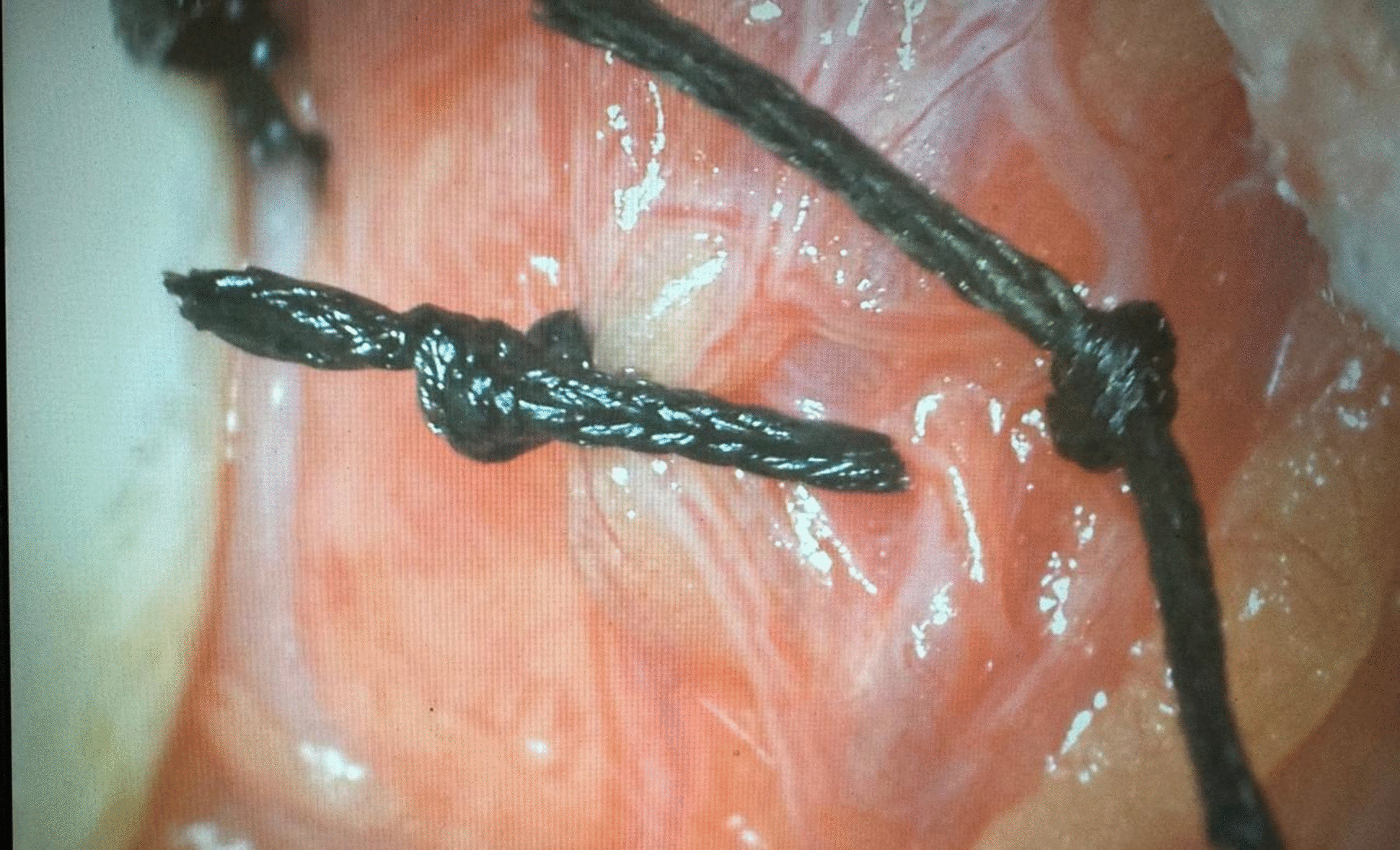
Ligation of the internal spermatic vein using microsurgical varicocelectomy (MSV) under optical magnification (× 8)

**Fig. 3:**
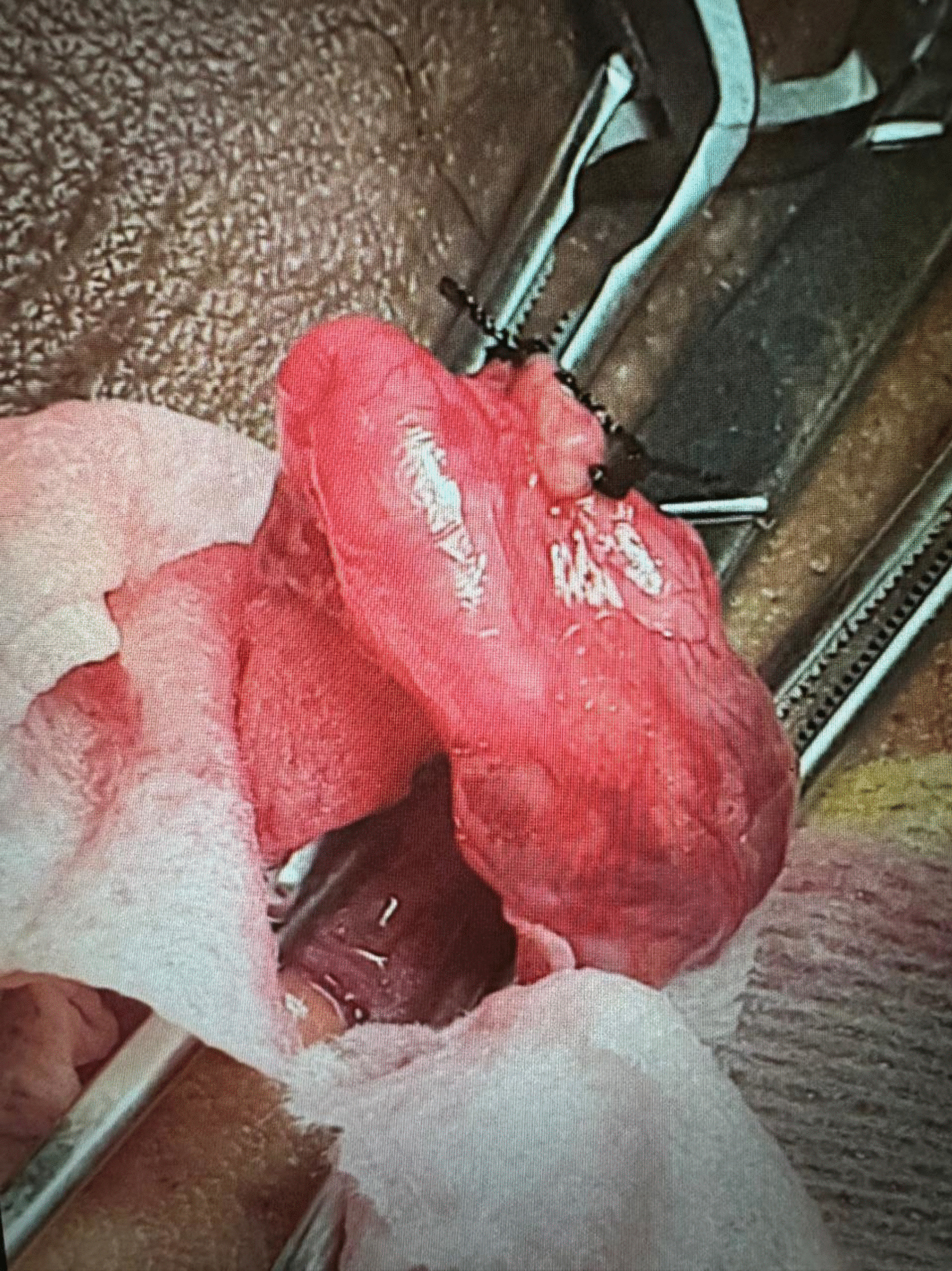
Injection of the internal spermatic vein by a sclerosant agent (1.5 to 3 mL of 3% aethoxisclerol mixed with 0.5 mL of air) using sclerotherapy. The cannulated vein is ligated with 4/0 polyglactin suture at the injection site in order to avoid leakage of the sclerosing agent. Also, any extracordal vein adjacent to the spermatic cord was ligated

**Fig. 4:**
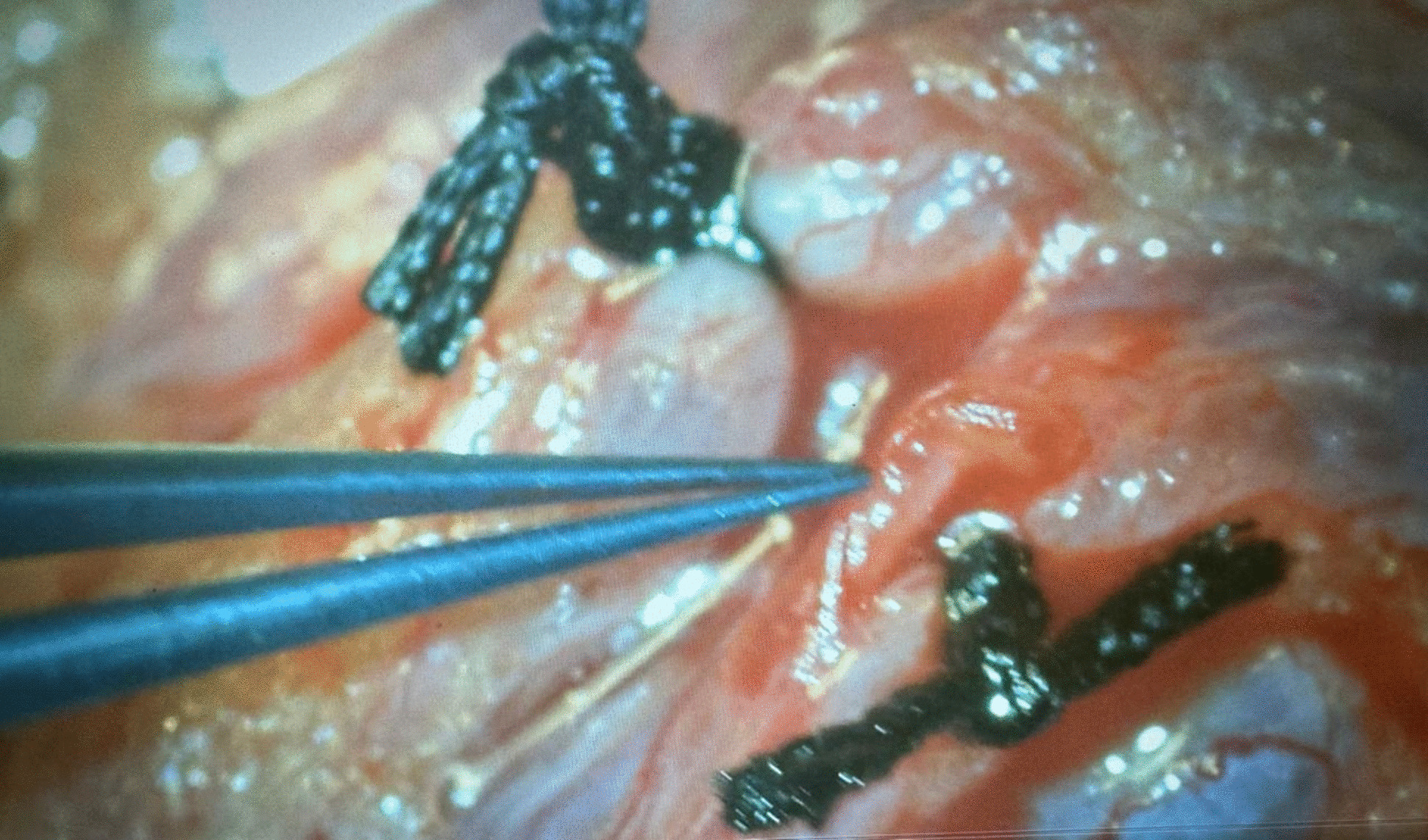
Mass ligation of the internal spermatic veins using Fisch technique after identifying the testicular artery under optical magnification (× 8)

A maximum venous diameter of 2.5 mm or more was considered diagnostic for a varicocele when measured with the patient in the upright position and during the Valsalva maneuver [19]. Reflux in the testicular veins lasting > 1 s with the patient standing and during the Valsalva maneuver was considered abnormal [19]. Routine preoperative labs including complete blood count, coagulation profile, liver enzymes, creatinine and random blood sugar were obtained. 34 patients (group I) underwent standard MSV technique which involved extensive dissections and ligations of adherent periarterial vein(s) under optical magnification (× 8) (Fig. 2) [10]. 22 patients (group II) underwent Colpi antegrade sclerotherapy of the temporarily occluded spermatic cord using 1.5 to 3 mL of 3% aethoxisclerol mixed with 0.5 mL of air (Fig. 3) [13]. 34 patients (group III) underwent Fisch technique that identified the testicular artery with utilizing × 8 magnification followed by mass ligation of the veins in the spermatic cord (Fig. 4) [15]. The operative time, recurrence rates, intra-operative and post-operative complications including hydrocele, testicular atrophy and wound infection were observed. Also, semen and sonographic parameters 3 months later were registered.

### Statistical methods

Data were coded and entered using the statistical package for the Social Sciences (SPSS) version 28. Data was summarized using mean and standard deviation for quantitative variables and frequencies (number of cases) and relative frequencies (percentages) for categorical variables. Comparisons between groups were done using analysis of variance with multiple comparisons post hoc test in normally distributed quantitative variables while non-parametric Kruskal–Wallis test and Mann–Whitney test were used for non-normally distributed quantitative variables*.*

For comparison of pre and post-operative measurements within each group paired t test was used in normally distributed quantitative variables while non-parametric Wilcoxon signed rank test was used for non-normally distributed quantitative variables. For comparing categorical data, Chi square (2) test was performed. Exact test was used instead when the expected frequency is less than 5. Normality of data was tested using normality plots and Shapiro Wilk test. P-values less than 0.05 were considered as statistically significant.

### Sample size

Using power sample size calculator for non-inferiority intervention study; with 0.05 alpha error and power of the study, enrolment ratio of 3, 0.80, CI of 95%, non-inferiority margin of 0.2. According to literature, the average operating times of Fisch’s artery-sparing technique with MSV for group I and group II were 94 ± 6.9 and 44.5 ± 8.4 min, respectively [20]. Also, the total recurrence rates and semen analysis postoperative motility improvement for group I and group II were 4.5%, 2% and 41.6%, 47.2%, respectively [20]. The operative time of subinguinal approach combined with antegrade intraoperative sclerotherapy of venous vessels was 25 min [13]. Thus, the required sample size calculated to compare the outcome of the three techniques was 66 patients to cover follow up period (22 in each group).

## Results

The sociodemographic characteristics were shown in Table 1. Regarding the baseline vein diameter among the study groups, there was insignificant difference on the left side among the studied groups (Table 2).

**Table 1:** Sociodemographic characteristics and clinical examination findings of the participants

		MSV (*N* = 34)^a^		Sclerotherapy (*N* = 22)		Fisch technique (*N* = 34)		*P* value
		Mean	SD	Mean	SD	Mean	SD	
Infertility duration (years)		2.60	± 0.57	2.52	± 0.65	2.76	± 1.02	0.498^d^
Age (years)		29.21	± 2.95	28.09	± 3.75	29.79	± 3.45	0.183^d^
		MSV		Sclerotherapy		Fisch technique		*P* value
		Count	%	Count	%	Count	%	
infertility type	Primary	10	29.4%	9	40.9%	13	38.2%	0.624^d^
	Secondary	24	70.6%	13	59.1%	21	61.8%	
Special habits	Smoker	22	64.7%	9	40.9%	22	64.7%	0.143^d^
	non smoker	12	35.3%	13	59.1%	12	35.3%	
RT^b^ testis size (ml)	normal	33	97.1%	22	100%	34	100%	1^d^
	moderate	1	2.9%	0	0.0%	0	0.0%	
LT^c^ testis size (ml)	normal	22	64.7%	15	68.2%	26	76.5%	0.558^d^
	moderate	12	35.3%	7	31.8%	8	23.5%	
RT^b^ cord exam	Grade II	34	100%	22	100%	34	100%	––-
LT cord exam	Grade II	30	88.2%	18	81.8%	30	88.2%	0.790^d^
	Grade III	4	11.8%	4	18.2%	4	11.8%	
RT^b^ cord post-operative	negative	32	94.1%	21	95.5%	32	94.1%	1^d^
	recurrent	2	5.9%	1	4.5%	2	5.9%	
LT^c^ cord post-operative	negative	32	94.1%	21	95.5%	32	94.1%	1^d^
	recurrent	2	5.9%	1	4.5%	2	5.9%	

N.B ^a^ *MSV* microsurgical varicocelectomy; ^b^ *RT* right; ^c^ *LT* left; ^d^ *p* value was calculated using Chi square (2) test

**Table 2:** Baseline semen parameters and scrotal duplex findings

	MSV (*N* = 34)^a^		Sclerotherapy (*N* = 22)		Fisch technique (N = 34)		^d^ *P* value
	Mean	SD	Mean	SD	Mean	SD	
RT^b^ testicular volume (ml)	14.76	± 1.39	14.73	± 1.64	14.62	± 1.30	0.909
Lt^c^ testicular volume (ml)	12.71	± 1.57	12.23	± 1.63	13.24	± 1.28	0.047
RT^b^ vein diameter (mm)	2.68	± 0.16	2.52	± 0.23	2.82	± 0.16	0.042
Lt^c^ vein diameter (mm)	3.25	± 0.24	3.35	± 0.37	3.34	± 0.24	0.308
RT^b^ reflux duration (seconds)	1.36	± 0.34	1.26	± 0.32	1.38	± 0.37	0.408
Lt^c^ reflux duration (seconds)	1.74	± 0.39	1.70	± 0.35	1.65	± 0.30	0.599
Semen volume (ml)	3.10	± 0.81	2.77	± 0.70	3.05	± 0.81	0.293
Sperm concentration (Million/ml)	8.40	± 2.90	10.36	± 3.38	8.14	± 2.96	0.022
Total sperm count (million)	25.65	± 10.10	28.32	± 10.95	24.76	± 11.64	0.364
Total sperm motility (%)	21.32	± 8.19	24.64	± 9.50	22.85	± 8.29	0.369
Progressive sperm motility (%)	12.53	± 3.98	13.95	± 6.76	14.06	± 6.45	0.491
total motile count/ejac (million)	5.12	± 2.23	7.07	± 3.90	5.80	± 3.86	0.197
Progressive motile count/ ejac (million)	3.10	± 1.45	3.86	± 2.40	3.52	± 2.61	0.708
Sperm normal forms (%)	2.24	± 0.61	2.36	± 0.58	2.35	± 0.49	0.603

N.B ^a^ *MSV* microsurgical varicocelectomy; ^b^ *RT* right; ^c^ *LT* left; ^d^ *p* value was calculated using analysis of variance with multiple comparisons post hoc test in normally distributed quantitative variables while non-parametric Kruskal–Wallis test and Mann–Whitney test were used for non-normally distributed quantitative variables

However, the difference in the baseline right vein diameter was statistically significant among the studied groups. Additionally, there were insignificant differences between the study groups in terms of the reflux duration bilaterally, baseline total sperm count, total sperm motility, progressive sperm motility, total motile count/ejaculate, progressive motile count/ejaculate and sperm abnormal forms (Table 2). However, the baseline sperm concentration was significantly different (P = 0.022) (Table 2). Remarkably, the patients who underwent MSV showed the longest operative time (66.29 ± 2.78 min), followed by Fisch technique (56.94 ± 3.07 min) then sclerotherapy (55.45 ± 1.99). Thus, the difference in the operative time between the three techniques was statistically significant (*P* < 0.001). Regarding the postoperative right vein diameter, MSV group showed the largest diameter (2.14 ± 0.15 mm), followed by Fisch technique (2.13 ± 0.15 mm) then sclerotherapy (1.75 ± 0.42 mm) (Table 3). Regarding the postoperative left vein diameter, MSV group showed again the largest diameter (2.17 ± 0.21 mm), followed by Fisch technique (2.14 ± 0.15 mm) then sclerotherapy (1.75 ± 0.42 mm) (Table 3). Moreover, the patients who underwent sclerotherapy showed the highest progressive sperm motility percent (25.27 ± 4.00%), followed by Fisch technique (21.56 ± 7.30%) then MSV group (19.85 ± 6.33%) (Table 3). Post hoc pair wise comparisons revealed that sclerotherapy and Fisch technique had a significantly higher effectiveness in reducing the operative time than MSV (Table 4). Additionally, it revealed that sclerotherapy technique had a significantly higher effectiveness in reducing the postoperative vein diameter measurement compared to MSV and Fisch technique (Table 4). Post hoc pair wise comparisons revealed that sclerotherapy technique had a significantly higher effectiveness in improving the postoperative progressive sperm motility percent compared to MSV (Table 4).

**Table 3:** Post-operative semen parameters and scrotal duplex findings

	MSV (*N* = 34)^a^		Sclerotherapy (*N* = 22)		Fisch technique (*N* = 34)		^d^ *P* value
	Mean	SD	Mean	SD	Mean	SD	
RT^b^ testicular volume (ml)	14.74	± 1.44	14.73	± 1.64	14.65	± 1.32	0.964
Lt^c^ testicular volume (ml)	12.71	± 1.57	12.23	± 1.63	13.24	± 1.28	0.047
RT^b^ vein diameter (mm)	2.14	± 0.15	1.75	± 0.42	2.13	± 0.15	< 0.001
Lt^c^ vein diameter (mm)	2.17	± 0.21	1.75	± 0.42	2.14	± 0.15	< 0.001
RT^b^ reflux duration (seconds)	0.06	± 0.24	0.05	± 0.21	0.06	± 0.24	0.972
Lt^c^ reflux duration (seconds)	0.06	± 0.24	0.05	± 0.21	0.06	± 0.24	0.972
Semen volume (ml)	2.85	± 1.03	2.78	± 0.95	2.88	± 1.04	0.961
Sperm concentration (Million/ml)	16.56	± 3.63	15.78	± 3.24	15.18	± 3.25	0.251
Total sperm count (million)	47.26	± 20.18	40.86	± 21.65	43.81	± 19.67	0.441
Total sperm motility (%)	39.38	± 2.73	40.32	± 1.89	39.18	± 3.47	0.327
Progressive sperm motility (%)	19.85	± 6.33	25.27	± 4.00	21.56	± 7.30	0.008
total motile count/ejac (million)	18.71	± 8.36	16.45	± 8.83	17.16	± 7.97	0.470
Progressive motile count / ejac (million)	9.44	± 5.25	10.41	± 5.63	9.99	± 6.67	0.680
Sperm normal forms (%)	3.53	± 0.61	3.59	± 0.59	3.56	± 0.61	0.933

N.B ^a^ *MSV* microsurgical varicocelectomy; ^b^ *RT* right; ^c^ *LT *left; ^d^ *p* value was calculated using analysis of variance with multiple comparisons post hoc test in normally distributed quantitative variables while non-parametric Kruskal–Wallis test and Mann–Whitney test were used for non-normally distributed quantitative variables

**Table 4:** Post hoc pair wise comparisons of significant variables

		MSV^a^ (N = 34)	Sclerotherapy (N = 22)	Fisch technique (N = 34)
Operative Time (minutes)	MSV		< 0.001	< 0.001
	Sclerotherapy	< 0.001		0.150
	Fisch technique	< 0.001	0.150	
RT^b^ vein diameter post-operative (mm)	MSV		< 0.001	1.000
	Sclerotherapy	< 0.001		< 0.001
	Fisch technique	1.000	< 0.001	
LT^c^ vein diameter post-operative (mm)	MSV		< 0.001	1.000
	Sclerotherapy	< 0.001		< 0.001
	Fisch technique	1.000	< 0.001	
Progressive sperm motility (%)	MSV		0.006	0.794
	Sclerotherapy	0.006		0.099
	Fisch technique	0.794	0.099	

N.B ^a^ MSV = microsurgical varicocelectomy; ^b^ RT = right; ^c^ LT = left; p value was calculated using Bonferroni test

## Discussion

The present study had shown insignificant differences between the studied patients in terms of baseline total sperm count, total sperm motility, progressive sperm motility, total motile count/ejaculate, progressive motile count/ejaculate and sperm normal forms apart from sperm concentration and the baseline right vein diameter. As the baseline sperm concentration showed the highest concentration in the sclerotherapy group while the largest baseline right vein diameter was observed in the Fisch technique group. This finding could be seen in alignment with Feng et al. (2022) who revealed insignificant differences between the studied patients in MSV and sclerotherapy groups regarding preoperative semen parameters [12]. Furthermore, the present study had revealed marked improvement within each group regarding post-operative sperm concentration, total sperm count, total sperm motility, total motile count/ejaculate, and progressive motile count/ejaculate and sperm abnormal forms. However, such differences between the 3 groups were insignificant. The present study showed that there was a significant reduction in the measurement of the post-operative vein diameter in patients who underwent sclerotherapy compared to those who underwent MSV and Fisch techniques. This finding could be explained by the fact that the sclerosant agent injected in the veins cause subintimal damage followed by collapse of the veins [21]. Interestingly, the current study had revealed a significant improvement in the post-operative progressive sperm motility percent in patients who underwent sclerotherapy compared to those who underwent MSV. This could be explained by the fact that the largest baseline left vein diameter was observed in the sclerotherapy group. Also, baseline right vein diameter was > 2.5 mm in the sclerotherapy group.

Similarly, Shiraishi et al. (2001) stated that patients with a testicular vein diameter > 2.5 mm had a significantly higher improvement index in sperm parameters after varicocelectomy than those with a testicular vein diameter < 2.5 mm [22]. In the same context, Alaymen (2006) recommended varicocelectomy in cases of testicular vein diameter > 2.5 mm [23]. Our findings regarding the efficacy of sclerotherapy in infertile patients with Vx were in agreement with [12, 24, 25]. Conversely, the current study had revealed that the patients who underwent MSV showed a statistically significant difference between baseline and post-operative sperm concentration, total sperm count, total sperm motility, progressive sperm motility, total motile count/ejaculate, progressive motile count/ejaculate and sperm normal forms. This finding could be seen in line with previous studies [12, 26–30]. In the same context, the European association of urology stated that MSV was associated with the lowest incidence of complications and recurrences together with the highest spontaneous pregnancy rate [31]. Remarkably, MSV had demonstrated significant improvement in sperms concentration compared to the other two techniques. Nevertheless, there is no explanation for such significant improvement in the sperms concentration 3 months after MSV according to the authors’ point of view. Consistently, how varicocele could affect male fertility and whether varicocelectomy could ameliorate male infertility or not, were not fully understood [32]. Moreoever, the beneficial effect of varicocelectomy on semen parameters and fertility status is still questionable by some clinicians [33–35]. Additionally, the current study had demonstrated that the patients who underwent Fisch technique revealed statistically significant differences between baseline and post-operative veins diameter, reflux durations, sperm concentration, total sperm count, total sperm motility, progressive sperm motility, total motile count/ejaculate, progressive motile count/ejaculate and sperm abnormal forms.

In a similar trend, Elahwany (2018) revealed statistically significant differences between baseline and post-operative sperm abnormal forms and sperm concentration and sperm motility among patients who underwent Fisch technique [20]. Noteworthy, the marked improvement in the semen parameters observed after 3 months following the 3 different techniques in the studied cases can be attributed to the effect of varicocelectomy in decreasing the sperm DNA fragmentation index as well as seminal plasma malondialdehyde that was revealed by Cannarella et al. (2024) [36]. In contrast, Fabiani et al. (2022) failed to demonstrate significant improvement in sperm count in patients who underwent surgical ligation and sclerotherapy [37]. This also agrees with Elahwany (2018) who noted a statistically insignificant difference between patients in Fisch technique group and those in MSV group regarding pre-operative and postoperative sperm count improvement [20]. Regarding the total recurrence rate in the present study, there was insignificant difference between Fisch technique and MSV after 3 months follow-up that could be seen in line with Elahwany (2018) and Hung et al. (2018) [20, 38]. In contrast, Colpi et al. (2006) revealed that the post operative recurrence of Vx after 3 months was lower than that in the current study [13]. To summarize our findings, the current study had shown that the 3 different techniques of varicocelectomy demonstrated significant improvement in different semen parameters. In the same context, a critical systematic review meta-analysis had provided a high level of evidence in favour of a positive effect of varicocelectomy to ameliorate semen parameters in infertile men with clinical Vx [39]. Contrariwise, some authors once again stated that the favourable effect of varicocelectomy still needed further elucidation [32] while others doubted a favourable effect of varicocelectomy on semen parameters [33–35].

### Limits of the study

There are several limitations that should be mentioned in the current study. The small sample size and the heterogeneity of the sample size as the baseline sperm concentration was highest in the sclerotherapy group. It should be mentioned that heterogeneity of the previous studies together with the remarkable high risk of publication bias towards studies with a positive data led to uncertain results. The underlying cause could be attributed to the higher chance of publishing manuscripts with statistically significant results compared to those with null results [40]. A phenomenon known as non-response bias owing to the refraining of the Investigators to submit their negative results [41]. Also, the largest left vein diameter was observed in the sclerotherapy group whereas the largest right vein diameter was observed in the Fisch technique. However, the prospective nature of the study added strength and reliability to it. Additionally, all the cases were consecutively recruited and suffered from clinical Vx with the diameter of the spermatic veins > 2.5 mm on both sides. Moreover, the short follow up period should be considered as a major limitation as extending the follow-up duration would provide more robust and reliable data. Nevertheless, the current study is one of the first that compares between 3 different techniques among challenging Vx cases with adherent periarterial veins. Furthermore, a recent published study compared between MSV and sclerotherapy after a mean follow up period of 5.1 months ± 0.57 for their patients which could be seen close to our period [21]. Finally, the inability to use the 6th edition of the WHO guidelines for semen analysis interpretation could also be considered as a limitation of the study [42]. However, it is worth mentioning that the 5th edition simplified the standardization of the semen analysis test through a structured step-by-step guidance to different conventional and extended semen tests [17].

Consistently, the 6th Edition of the WHO guidelines for semen analysis interpretation raised concerns that should be clarified for the surgeons who work in the male infertility field [43]. Given that the median sperms concentration in our study was relatively low, there might be a possibility that patients with sperms concentration below 5 million/mL may had been included in the study together with Y-chromosome microdeletion being not performed in the current study should be seen as another limitation. Henceforth, future studies with proper screen for such group of patients by performing karyotyping and Y-chromosome microdeletion are essentials for a more comprehensive exclusion of other potential causes of infertility [31].

## Conclusion

The 3 different techniques showed significant improvement in the semen parameters after 3 months in the studied patients. However, sclerotherapy technique showed a significantly higher effectiveness in improving the postoperative progressive sperm motility percent compared to MSV.

## Data Availability

The data that support the findings of this study are available from the corresponding author upon reasonable request.

## Abbreviations

Varicocele: Vx
MSV: Microsurgical varicocelectomy
